# Ventilation Positive Pressure Intervention Effect on Indoor Air Quality in a School Building with Moisture Problems

**DOI:** 10.3390/ijerph15020230

**Published:** 2018-01-30

**Authors:** Camilla Vornanen-Winqvist, Kati Järvi, Sander Toomla, Kaiser Ahmed, Maria A. Andersson, Raimo Mikkola, Tamás Marik, László Kredics, Heidi Salonen, Jarek Kurnitski

**Affiliations:** 1 Department of Civil Engineering, Aalto University, Rakentajanaukio 4, 02150 Espoo, Finland; kati.jarvi@aalto.fi (K.J.); sander.toomla@aalto.fi (S.T.); kaiser.ahmed@aalto.fi (K.A.); maria.a.andersson@helsinki.fi (M.A.A.); raimo.mikkola@aalto.fi (R.M.); heidi.salonen@aalto.fi (H.S.); jarek.kurnitski@aalto.fi (J.K.); 2 Department of Microbiology, University of Szeged, Közép Fasor 52, H-6726 Szeged, Hungary; mariktamas88@gmail.com (T.M.); kredics@bio.u-szeged.hu (L.K.); 3 Department of Civil Engineering and Architecture, Tallinn University of Technology, Ehitajate tee 5, 19086 Tallinn, Estonia

**Keywords:** ventilation, positive pressure, indoor air quality, mycobiota, indoor air questionnaire, moisture damage

## Abstract

This case study investigates the effects of ventilation intervention on measured and perceived indoor air quality (IAQ) in a repaired school where occupants reported IAQ problems. Occupants’ symptoms were suspected to be related to the impurities leaked indoors through the building envelope. The study’s aim was to determine whether a positive pressure of 5–7 Pa prevents the infiltration of harmful chemical and microbiological agents from structures, thus decreasing symptoms and discomfort. Ventilation intervention was conducted in a building section comprising 12 classrooms and was completed with IAQ measurements and occupants’ questionnaires. After intervention, the concentration of total volatile organic compounds (TVOC) and fine particulate matter (PM_2.5_) decreased, and occupants’ negative perceptions became more moderate compared to those for other parts of the building. The indoor mycobiota differed in species composition from the outdoor mycobiota, and changed remarkably with the intervention, indicating that some species may have emanated from an indoor source before the intervention.

## 1. Introduction

The success of moisture- and mold-damaged building repairs depends on many factors. Reaching a consensus about the necessary repair methods and schedule is not always simple, and the response to problems might therefore be delayed. Occupants’ symptoms and discomfort can have multiple causes, and detecting or treating them all is not always possible nor successful [1]. In cases where harmful impurities remain in the structures or crawlspaces after repairs, indoor air problems might still occur. It has been shown that negative pressure indoors may introduce harmful pollutants [2], e.g., fungal spores [3], which may cause adverse health effects among occupants [4]. Preventing infiltration of the possible impurities from the structures or surroundings, caused by high negative pressure, is crucial for maintaining a good indoor air quality (IAQ). In moisture damage repairs, a thorough renovation, including ventilation, is important [5].

Ventilation is strongly associated with perceived IAQ, health, and productivity [2,6]. The chosen ventilation strategy also significantly affects the indoor microbial community [7]. Indoor fungi can be useful indicators of IAQ, therefore a deeper understanding of their biology is of special importance [8]. Ventilation, as well as indoor temperature and humidity, have a significant impact on human perceptions and well-being [9,10]. Proper ventilation measurements should be an essential part of every IAQ investigation [11]. In air-tight buildings with a mechanical ventilation system, the balancing and controlling of the ventilation system and pressure differences are inevitable [12]. When evaluating the effect of ventilation on perceived IAQ, pressure difference measurements are important [13].

In cold-climate countries, keeping structures dry is a main issue, and ventilation design is strongly related to this objective. According to the National Building Code of Finland [14], pressure conditions should not generate moisture loads on structures and, at the same time, not assist pollutant transfer through the structures. A ventilation design is typically balanced, or the extract airflow rate is slightly higher than the supply, to prevent exfiltration of moist indoor air into the structures. However, in well-ventilated buildings, the indoor air is often dry, and school buildings are typically occupied only during normal office hours. In such buildings, the risk for condensation is improbable despite the pressure conditions.

This ventilation intervention study was conducted as part of the Finnish EURA- and TOXICPM-research projects concerning IAQ and ventilation in renovated school buildings and microbial toxin transport mechanisms. The building discussed in this study has a history of moisture damage repairs, yet occupants still report IAQ-related symptoms and discomfort. The building is awaiting new major repairs. Our main hypothesis is that positive pressure differences across the building envelope could prevent the infiltration of harmful chemical and microbiological agents into the indoor air of damaged structures, thus decreasing occupants’ perceptions of symptoms and discomfort.

The aims of our research project were to determine the effects of significant ventilation system changes on measured and perceived IAQ in a school building with unsolved indoor air-related problems, and to provide information about the applicability of moderate positive pressure in a well-ventilated building awaiting repairs. To test the hypothesis, chemical, microbial, and physical IAQ measurements, as well as occupant questionnaires, were conducted before and after ventilation system intervention.

## 2. Materials and Methods

### 2.1. Building Characteristics

The study was carried out in a comprehensive school in Vantaa, Southern Finland. The school was selected in cooperation with Vantaa Real Estate Center in the spring of 2016, based on its repair history and the fact that a reasonable and controllable part of the building could be isolated for implementing the positive pressure intervention without major ventilation system changes in the building. According to Vantaa Real Estate Center, several microbial and structural investigations had been made since 2004, and the building had experienced extensive moisture damage, air leakage, and ventilation system repairs during previous years. Yet IAQ-related or unspecific complaints from the occupants had continued. New structural repairs to the whole building took place during 2017.

**Figure 1:**
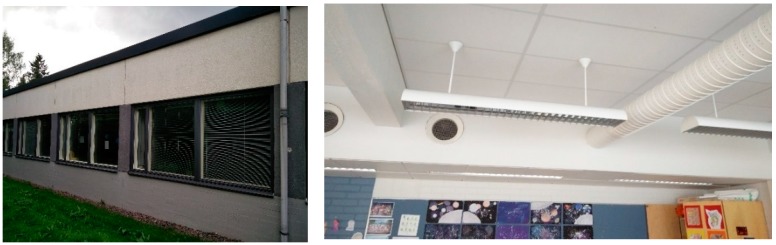
Studied building section, a typical supply air duct diffuser, and an extract air grille in a classroom.

The school was built in 1968 and fully renovated in 2003–2005. Approximately 700 students and over 50 staff members worked in the building. The mechanical supply and extract ventilation system with heat recovery in all classrooms and corridors was installed in 2002. The building was connected to the district heating network and had central heating systems with water radiators and thermostatic valves. Each classroom had 2–3 supply air duct diffusers and 1–2 extract air grilles. Airflow rates were adjusted by dampers in the main air handling unit and with regulation and measuring devices connected to each terminal device. The studied section of the building as seen from the outside, and the typical supply and extract terminal units of a classroom in the section, are shown in Figure 1.

**Figure 2:**
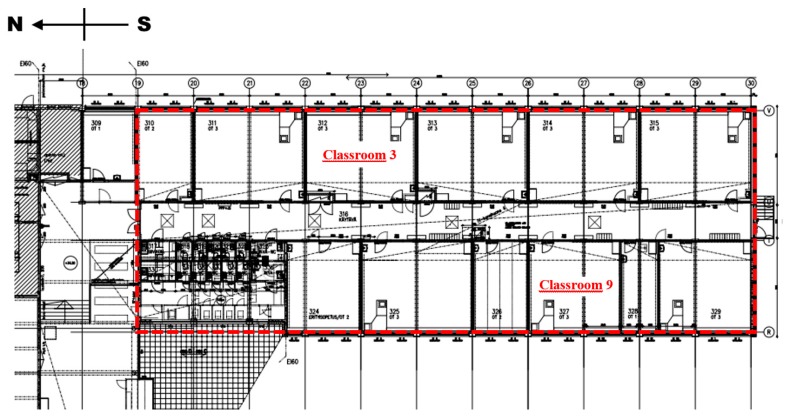
The ventilation intervention was conducted in the building section marked with red. Measurements were carried out in Classrooms 3 and 9.

The studied part of the building consisted of 12 classrooms, one corridor, six toilets, and a cleaning storage area, and was served by one air handling unit. Most of the classrooms were occupied by 20–25 humans for a few hours per day during the normal five-day school week. The layout of the studied part of the building and its orientation are presented in Figure 2. Measurements were carried out in two classrooms located at different sides of the studied building part: Classrooms 3 and 9. According to the surveys conducted by the occupational safety and health personnel, occupants had reported the most severe IAQ problems in those two classrooms.

### 2.2. Previous Investigations

Due to the prolonged IAQ-related complaints in the building, an indoor climate and structural investigation (report 24 August 2016 handed over to the Real Estate Center of the City of Vantaa) was conducted in the whole building by a certified consultant company, Sweco Finland Oy (Helsinki, Finland), during April 2016 [15], prior to this ventilation intervention research project.

In the base floor, tracer gas was used to detect the air flows possibly infiltrating from the ground. The air was found to infiltrate the room via wall/floor junctions and service entries, and the investigated classroom had negative pressure in relation to the ground filling. In outer wall tracer gas investigations, air leakages through the building envelope were found in the wall/floor junctions, window/wall junctions, and the supports for radiators and windowsills.

In the surface humidity detection of floorings, high humidity was detected widely in the studied building section, especially in the whole corridor area. During the cut measurement—where the flooring mat was cut and a relative humidity sensor was set under the flooring—the relative humidity (RH) under the flooring was normal, 28%, in Classroom 3. During drillhole measurements, a 16 mm hole was drilled to the concrete slab, measurement sensors were installed and sealed into the hole and read after stabilizing. High humidity was measured at all eight measurement spots (corridor and classrooms) with RH values between 87–95%. Nevertheless, simultaneous volatile organic compound (VOC) measurements in Classroom 3 did not show atypical material emissions referring to material deterioration (TVOC: 5 µg/m^3^, 2-ethyl-1-hexanol: 1 µg/m^3^). However, it was recommended that the surface flooring should be replaced with a more permeable material to avoid future problems.

In Classroom 3, the structures were opened for investigation. It was found that the outer wall structure did not follow the correct building design, i.e., the inner brick layer was covered by insulation and a vapor-tight concrete layer, which could lead to vapor condensation during the cold season. Material samples for microbiological analysis were taken from the structure’s opening. Microbial growth (fungi and bacteria) was found in the wooden structures of the window-frame fastening (Table 1). Actinobacteria and *Exophiala* * sp., which are indicators of moisture damage, were found in the mineral wool layer (Table 1). These findings verify the assumption that structures have been exposed to moisture and damaged because of incorrect building design. Microbial assessment of indoor air did not show increased microbial levels or moisture indicator microbes.

The investigation concluded that impurities from microbially damaged materials were possibly infiltrating indoor air, and might comprise a health risk. Infiltration is caused by air leakages and negative pressure, which is highest at night due to the continuously running extract air fans while supply air is shut down. Extensive structural repairs are needed in the lower parts of the outer wall to improve the building design as well as seal the air leakage routes.

### 2.3. Set-Up for Ventilation Intervention

The ventilation intervention was conducted in four phases: (a) airflow rates in the rooms were measured to determine the initial state of the ventilation; (b) the air distribution ductwork was fully balanced by an authorized company; (c) the air handling unit’s supply and extract fan speeds were adjusted to generate the desired 5–7 Pa positive pressure over the building envelope in each classroom; and (d) airflow rates in the rooms were re-measured, and pressure differences over the envelope were monitored during the entire set-up. Positive pressure across the exterior building envelope was maintained in the studied building section fairly well over the winter season, from August 2016 to May 2017.

The aim of generating low 5–7 Pa positive pressure was to prevent potential infiltration by harmful agents and avoid strong exfiltration of indoor air. In this ventilation intervention, it was possible to establish a positive pressure in the building by balancing the ventilation system and by adjusting fan speed control frequency in the air handling unit. The interzonal pressure differences of the studied building part were not measured, because the ventilation system was carefully balanced, and after the balancing all classrooms had practically the same supply and extract airflows eliminating potential pressure differences between classrooms. Each classroom had its own supply and extract air terminal units and classroom doors were typically kept close continuously, thus making the possible air mixing between rooms improbable.

Related IAQ measurements were conducted simultaneously with phases (a) and (d). Occupant perceptions were recorded, and microbial samples collected before the intervention and after five months of positive pressure. Moisture behavior of the structures during the positive pressure period was monitored, and will be reported in another journal article.

### 2.4. Measurement Methods

Some of the measurements were conducted in the entire building section under study; others were conducted in only the two classrooms with the most significant IAQ-related complaints from the occupants: Classrooms 3 and 9. The measured factors and measurement devices are presented in Table 2.

#### 2.4.1. Airflow Rate Measurements and Pressure Differences across the Building Envelope

Airflow rates were measured from each ventilation duct terminal unit in every room and in the corridor in the studied building section before and after balancing the ventilation, and after generating the positive pressure. Pressure differences across the building envelope were measured in Classrooms 3 and 9 continuously for one week before the ventilation intervention and for nine months after generating the positive pressure. A plastic tube with a copper core was placed outside by a window that was not normally open. A measurement device and a logger were placed inside near the window. The outdoor temperature was monitored simultaneously.

#### 2.4.2. Temperature, Relative Humidity, and CO_2_ Concentration of Indoor Air

Temperature (T), Relative Humidity (RH), and CO_2_ concentrations were measured in Classrooms 3 and 9 for a one-week period before the ventilation intervention, and for a nine-month period after the intervention. Measurement devices were placed on the teacher’s desk in the front of the room, away from the teacher’s breathing zone when seated and as close to the horizontal central area of the room as possible.

#### 2.4.3. Indoor Air Quality (IAQ) Measurements

VOCs were measured in Classrooms 3 and 9 and in the corridor before and after the ventilation intervention. TVOC and single compounds that had concentrations over 1 µg/m^3^ were analyzed. The VOC sampling and analyses were carried out according to ISO 16000-6 standard [17]. Air samples were taken from the central area of an empty, closed room, in the main working zone at a height of 1.5 m. Samples were collected in Markes International Ltd. (Llantrisant, UK) stainless steel tubes packed with Tenax TA (60/80 mesh) using GilAir Plus air sampling pumps (Sensidyne, St. Petersburg, FL, USA) at a flow rate of 200 mL/min for 40 min.

The samples were desorbed using a thermal desorption unit, TD-100 (Markes International Ltd.) and analyzed by a gas chromatograph, Clarus 580 (Perkin-Elmer Ltd., Beaconsfield, UK), equipped with a Clarus 600T (Perkin-Elmer Ltd.) mass selective detector. The VOCs were quantified by the scan (50–400 *m*/*z*) mode. Concentrations of TVOC and individual compounds were determined from TVOC area (*n*-hexane to *n*-hexadecane) and calculated as toluene equivalents. Concentrations of single compounds were also determined from the chromatogram before and after the TVOC area. In the case of such compounds, the quantitative result was indicative. Reference compounds and the NIST 2011 Mass Spectral Library automated mass spectral deconvolution and identification system (AMDIS) was used for identification. The detection limit was 0.2 µg/m^3^ (not included in sum concentration).

The formaldehyde concentration of indoor air was measured using an FM-801 formaldehyde meter (GrayWolf Sensing Solution, Sheldon, LA, USA), and carbon monoxide (CO) was measured with an electrochemical sensor with a TG-501 probe using an AdvancedSense meter (GrayWolf Sensing Solution). Fine particulate matter (PM_2.5_) was measured using a MIE pDR-1500 (Thermo Fisher Scientific, Franklin, MA, USA) nephelometer equipped with a PM_2.5_ size-selective inlet cyclone. Formaldehyde and PM_2.5_ were measured continuously for a one-week period before and after ventilation intervention, while CO was measured for a one-week period after the intervention. Measurements were conducted in Classroom 3, since only a single measurement device was available for these measurements.

Formaldehyde, CO, and PM_2.5_ measurement devices were placed in the back of the room, at a height of 1.5 m, as close to the central area of the room as possible. Inviolability of the devices had to be considered since the measurements were carried out for several days in a room occupied by children.

#### 2.4.4. Characterization of the Mycobiota in the Indoor Dust

The mycobiota of indoor dust was collected from the studied building section’s extract air filter, as well as from the settled dust collected from Classrooms 3 and 9. Reference dust was collected from the outdoor air. Hay barn dust was also studied as a reference sample of rich, natural microbial environment. Characterization of the mycobiota was accomplished in three stages:

1. Sampling of dust from the extract air filter, air, and surfaces above floor level.

Material samples from the extract air filter were collected in sterile plastic bags. Pieces of the filter material (ca. 1 cm × 1 cm) were spread on malt extract agar (MEA) plates (Malt extract 15 g: Sharlab, Barcelona, Spain; agar 12 g: Amresco, Solon, OH, USA, in 500 mL of H_2_O). Dust samples were wiped into a clean plastic bag (Minigrip: Amerplast, Tampere, Finland) from ca. 30 × 30 cm^2^ surfaces 1–2 m above floor level. The dust (ca. 10 mg) was spread with a sterile cotton swab on MEA plates. Air samples were collected with MEA fallout plates that were kept open for an hour. Culture plates were inoculated, sealed, and cultivated at 22 °C for four weeks.

2. Rapid toxicity screening of single colonies with boar sperm and somatic cell lines.

For initial toxicity screening, 10–20 mg of biomass (wet weight) from each colony of the original culture plates was looped into 0.2 mL of ethanol and heated in a water bath for 10 min at 80 °C. The obtained ethanolic lysates were exposed to porcine spermatozoa and kidney tubular epithelial cells (PK-15, Finnish Food Safety Authority, EVIRA, Helsinki, Finland). The lysate was considered toxic when 2.5 vol % decreased boar sperm motility, or 5 vol % decreased proliferation of PK-15 cells by >50% compared to the sham exposed control. Boar sperm motility inhibition assay (BSMI) measuring motility inhibition (i.e., inability to respond to induction of motility in resting sperm cells exposed for one day at room temperature) is described in [18]. The inhibition of cell proliferation (ICP) assay with PK-15 cells and the determination of EC_50_ concentrations followed the methods described by [19]. The colonies that displayed toxicity were streaked pure and identified to genus or species level.

3. Characterization and identification of the fungal isolates.

Fungal colonies were grouped into 12 morphotypes based on colony morphology on MEA, ability to grow at 37 °C, light microscopy of the conidia and conidiophores, and responses in the two toxicity assays, BMSI and ICP. The isolates were compared to the identified strains from our strain collection, HAMBI, or identified according to [20]. Representatives for the morphotypes were identified according to their ITS sequences [21].

#### 2.4.5. Indoor Air Questionnaire

Occupants’ indoor air-related symptoms and discomfort were recorded with the standardized Indoor Air Questionnaire of the Finnish Institute of Occupational Health (FIOH) twice during the research. The questionnaire was based on the Örebro Indoor Climate Questionnaire MM40 [22].

The questionnaire consists of four different foci: (1) the work environment; (2) the work arrangements; (3) the employees’ allergy history; and (4) work-related symptoms. The questionnaire asked the respondents to recall environmental problems (draft, dry or stuffy air, etc.) that had occurred during the past three months.

Staff members of the whole school were requested to participate in the questionnaire, and the principal of the school arranged for the delivery of the questionnaires at the workplace. Staff members had two weeks to respond to the questionnaire. FIOH collected and reported the answers.

Potentially significant differences between the two questionnaires were analyzed at Aalto University by the SPSS statistical software (SPSS Finland Oy, Espoo, Finland), with a chi-squared test. The main interest was to determine whether symptoms supposedly related to impurities from the structures could be reduced by converting infiltration to exfiltration in the studied building section.

## 3. Results and Discussion

### 3.1. Airflow Rate Measurements and Pressure Differences across the Building Envelope

Total airflow rates before and after the ventilation intervention are shown in Table 3.

Before balancing, the airflow rates were found to be heavily unbalanced in most classrooms, as well as in the corridor and in the toilets (Table 3). According to Finnish regulations, the acceptable deviation at room level and system level is 20% and 10%, respectively. At classroom level, the supply airflow rates were within ±20% of the designed values, with two significant exceptions of −36% (Classroom 7) and +57% (Classroom 8). Almost all the classrooms had extract airflow rates over 20% higher than the designed values. The corridor extract was 18% lower than the designed values. The extracts from the toilet and storage units were 55% lower than the designed values, which was found to be caused by the wrong rotation direction of the fan. At a system level, the total extract airflow rate was 18% higher than the designed values. After generating the positive pressure, all classrooms except from Classrooms 4 and 8 received more supply than extract air. Airflow rates were measured as spot checks from some classrooms in spring 2017, and had been maintained at the required level.

**Figure 3:**
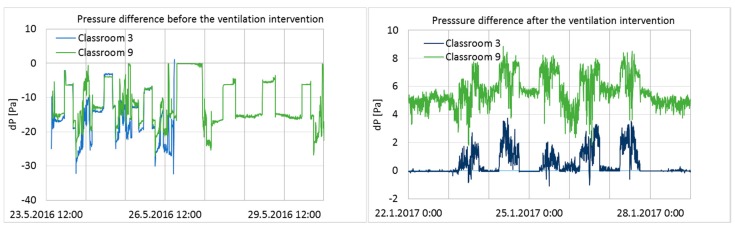
One-week pressure difference across the building envelope in Classrooms 3 and 9 before and after the ventilation intervention. In Classroom 3, the results before intervention were recorded only for half of the week because of dysfunction of the measurement device.

Pressure differences across the building envelope from one week before and after the intervention in Classrooms 3 and 9 are shown in Figure 3.

**Figure 4:**
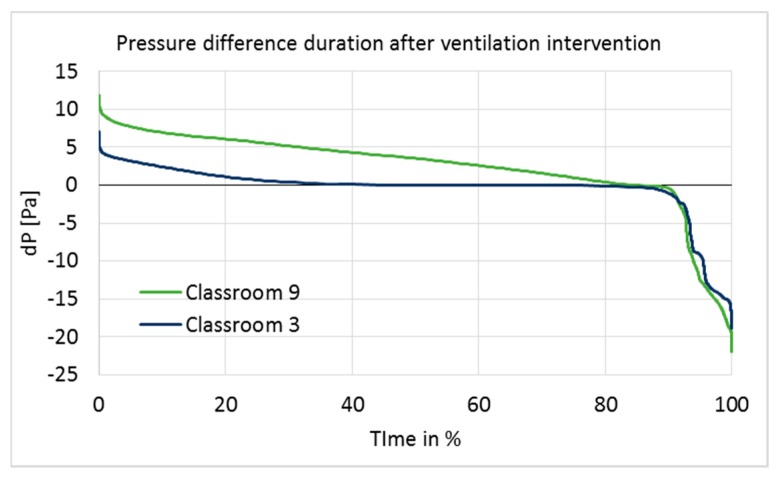
Duration of pressure differences across the envelope in Classrooms 3 and 9 during nine months after ventilation intervention.

Before the ventilation balancing, pressure differences fluctuated within a large range during a one-week period, and both measured rooms had a significant level of negative pressure. After the ventilation balancing, the pressure differences across the envelope were moderately stable and positive, and fluctuation was minimal compared to the conditions before the intervention (Figure 3). Pressure differences over the entire ventilation intervention period are shown as cumulative frequency curves (duration curves) in Figure 4.

Positive pressure was maintained rather well in Classroom 9, but poorly in Classroom 3 (Figure 4). In Classroom 9, the pressure difference was at the optimal level of 5–7 Pa for 23% of the measurement time, and over 0 Pa for 84% of the time. In Classroom 3, it was over 0 Pa for 45% of the time; thus, most of the time, Classroom 3 had a pressure difference very close to 0.

According to the set-up design, positive pressure should have been maintained continuously throughout the entire intervention period. However, the results showed that the ventilation system was turned off at night by the school maintenance or by the automatic remote-control system during school holidays; it was also turned off during other short-term periods for unknown reasons. The longest holiday period was the Christmas holiday, between 21 December 2016 and 7 January 2017, during which time the ventilation system was clearly turned off every day from 4 p.m. to 6 a.m., and the pressure difference became highly negative. Negative pressure differences during that period were significant (up to −18 and −22 Pa in Classrooms 3 and 9; average pressure difference −7.9 and −8.4 Pa), generating unwanted effects on the ventilation intervention.

The building was unoccupied during holiday periods; thus, pressure fluctuation did not have a direct impact on the occupants. Furthermore, if the Christmas holiday period is ignored, then the pressure difference becomes positive (>0) for 46% (Classroom 3) and 88% (Classroom 9) of the time, which does not make a significant difference to the duration of the pressure difference on a longer period of time. However, possible impurities inside the structures may have infiltrated indoors during holiday periods, which would have influenced the indoor conditions and might have affected the user questionnaire responses accordingly. Therefore, holiday times, especially the Christmas holiday, were not excluded from the data.

**Figure 5:**
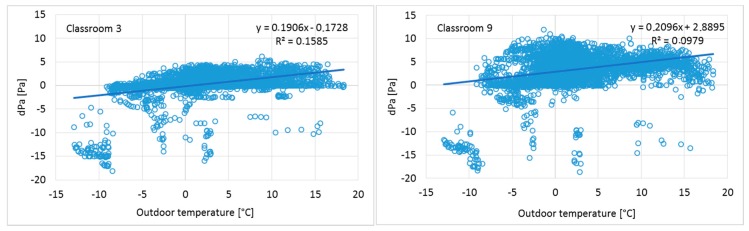
Relation between outdoor temperature and pressure difference across the envelope in Classrooms 3 and 9 with air handling unit without density-corrected airflow rates.

The measured results show an increase of positive pressure when the outdoor temperature increases (Figure 5), and a corresponding decrease when the outdoor temperature drops. This has provided an additional challenge for positive pressure as adjustments were done during warm season. These results indicate typical phenomena of heat recovery ventilation units: During the cold season, the mass flow of constant volume extract fans becomes higher because the temperature after heat recovery is lower and the density of the air is correspondingly higher. At the same time, the supply air fan operates at stable air temperature conditions and the mass flow does not change. To avoid this phenomenon, an air handling unit with density-corrected air volume flow rate control should be used to maintain a continuous positive pressure in a building.

In a cold climate, water vapor condensation and moisture accumulation inside structures are seen as potential risks when pressure differences across the envelope are positive. To evaluate the risk of condensation inside the structures, excessive moisture in Classrooms 3 and 9 was calculated as the difference of measured absolute humidity of indoor and outdoor air, before the positive pressure period began. To monitor the possible moisture condensation risk in the structures during the ventilation intervention, T and RH probes were installed inside the structures at the most probable leakage paths, as well as in a reference room. According to humidity measurements, the structures were dry, indicating that this moisture risk was not present because of the modest humidity load in well-ventilated classrooms [23].

### 3.2. Temperature, Relative Humidity, and CO_2_ Concentration of Indoor Air

**Figure 6:**
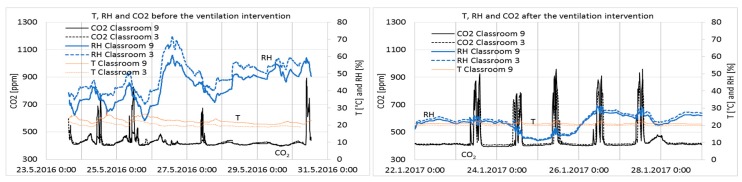
Temperature, relative humidity, and CO_2_ concentration in Classrooms 3 and 9 one week before and after the ventilation intervention.

Results from one week before and after ventilation intervention are shown in Figure 6.

**Figure 7:**
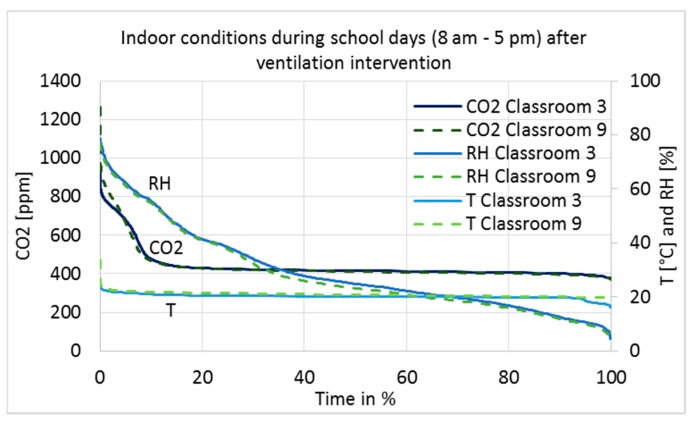
CO_2_ concentration, relative humidity, and temperature duration in Classrooms 3 and 9 during school’s occupancy hours, from 8 a.m. to 5 p.m. on Monday–Friday, measured continuously from 24 August 2016 to 9 May 2017 after ventilation intervention was conducted.

The measurement results after the ventilation intervention are shown as a duration curve in Figure 7, and average, minimum, and maximum values are described in Table 4. Only results during school working hours, from 8 a.m. to 5 p.m., are shown to describe conditions during occupancy.

The maximum limit value for CO_2_ concentrations in Category I of the Finnish Classification [24] is 750 ppm; the limit value in Category II is 900 ppm, while in Category III it is 1200 ppm. The stability of the conditions has to be 95% (Category I) and 90% (Category II). Category III meets the minimum requirements of the Finnish regulation, Category II is defined as good and Category I as best possible IAQ. The levels of CO_2_ concentrations before and after the intervention were in Category I (Figure 7 and Table 4).

The RH of the classrooms closely followed the absolute humidity of the outdoor air, i.e., values are very low in cold winters and reach 75% on summer days. In Category I [24], it is recommended that RH should not drop for long periods below 20%; no recommendations exist for the other categories. It can be seen in Classrooms 9 and 3 that RH was below 20% for 31% and 26% of the school hours, respectively (Figure 7). Such dry air causes irritation of the respiratory tract, eyes, and skin [25]. Indirectly, temperature and RH affect human perceptions of air quality and emissions from building materials [26,27,28] with negative effects associated to higher temperature and RH values. From this point of view, there are some arguments for humidification during cold winter time, but as far as Category II spaces are considered, it is not needed. Contra argument to humidification is to keep the ventilation systems simple and not equipped with humidification components due to cost, hygienic and maintenance issues.

Temperatures were very stable and slightly higher in Classroom 9 (Table 4), which can be explained by the location of the room on the western side of the building.

### 3.3. Indoor Air Quality (IAQ) Measurements

TVOC, VOC, formaldehyde, and CO measurement results are shown in Table 5.

TVOC values decreased 21–55% in the classrooms and in the corridor after ventilation intervention (Table 5). This result was statistically significant at a 95% confidence interval (*p* = 0.045). Besides positive pressure, no other changes were undertaken; therefore, the only known explanation for the decreased concentrations is the positive pressure and infiltration mainly converted to exfiltration. Concentrations were very low in general, compared to the national threshold values [29], indicating good ventilation and off-gassing of materials.

Concentrations of single VOCs were 1–4 μg/m^3^, which is fairly below action level. TVOC concentrations consist mainly of compounds with concentrations below 1 µg/m^3^. In addition to the general limit value for single compounds, national limit values for the following VOCs are 2,2,4-trimethyl-1,3-pentanediol di-isobutyrate (TXIB) (10 µg/m^3^), 2E1H (10 µg/m^3^), naphthalene (10 µg/m^3^), and styrene (40 µg/m^3^) [29]. In these samples, only 2E1H was shown, but only as low concentrations from 1 to 3 µg/m^3^ (Table 5).

For formaldehyde, the maximum allowed value is 50 (annual average) or 100 µg/m^3^ (30 min average), while for CO it is 7 mg/m^2^ (momentary concentration) [29]. The formaldehyde concentration before and after the ventilation balancing was below 10 ppb (equivalent to approx. 12 µg/m^3^), which is the detection limit of the meter. After balancing, there was a concentration of 12–26 ppb during two hours on one day, which was probably due to some specific action in the classroom, e.g., art lecture. The CO concentration varied from 0.3 to 1.3 mg/m^2^.

**Figure 8:**
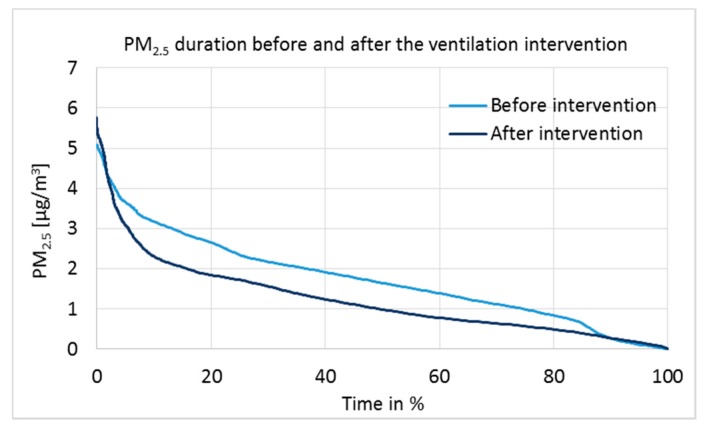
Duration curves of particulate matter 2.5 µm before and after the ventilation intervention in Classroom 3 during one week.

Particulate matter 2.5 µm measurement results are presented as a duration curve in Figure 8, and parameters for statistical analysis are given in Table 6.

The limit value for the PM_2.5_ mean concentration following 24 h in indoor air is 25 µg/m^3^ [29]. All known PM_2.5_ sources in the studied building are outdoor sources; for example, traffic and burning products. PM_2.5_ concentrations were very low before and after the intervention (Figure 8). However, tested by the independent two-sample *t*-test (Table 6), the difference in medians was statistically extremely significant at the 100% confidence interval (*p* = 0.000, 2-tailed). Daily actions can affect the PM_2.5_ concentrations temporarily; but due to the one-week measurement time, the results are reliable and show a clear correlation with the positive pressure.

### 3.4. Characterization of the Mycobiota in the Indoor Dust

**Figure 9:**
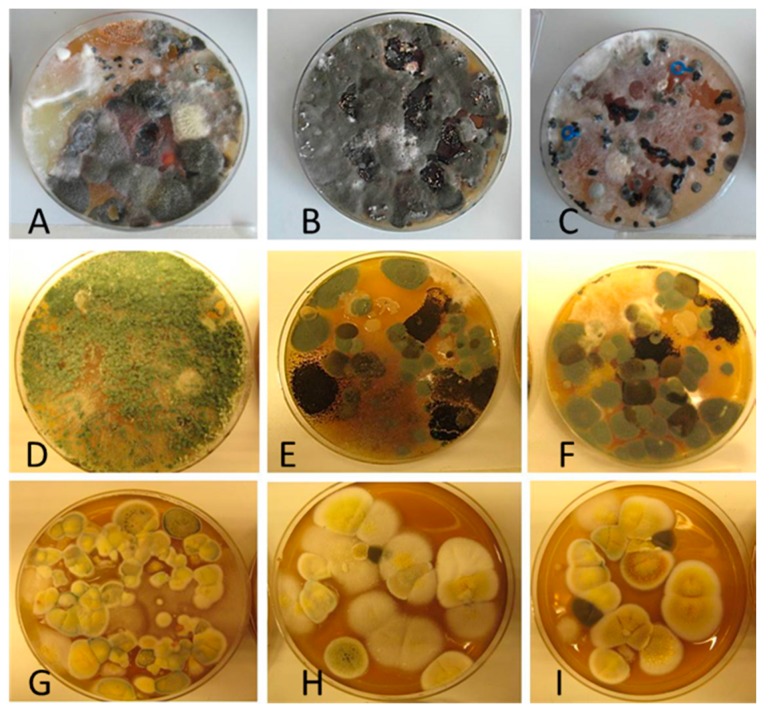
Fungal colonies sampled from the outdoor air and from the extract air filter dust before and after the ventilation intervention. The outdoor air and dust contained black *Curvularia*-like colonies (**A**–**C**). Extract air filter sampled before the ventilation intervention contained green *Trichoderma* colonies (**D**), and green *Penicillium* and black *Aspergillus* colonies (**E**,**F**). Extract air filter sampled after the ventilation intervention contained yellow *Penicillium* colonies and yellow *Aspergillus* colonies (**G**–**I**).

Diversity and the pathogenic and toxigenic potential of the mycobiota cultivable from indoor dust were characterized. Dust from extract filters and surfaces above floor levels were sampled and cultivated before and after the ventilation intervention. Outdoor dust and air were sampled as references. The fungal colonies are shown in Figure 9.

The fungal colonies representing the dominant morphotypes after four weeks of incubation were characterized to genus or species level (Table 7). The results in Table 8 show that *Rhizopus* sp. strains unable to grow at 37 °C were the most commonly isolated fungi in filter dust and settled dust collected before and after ventilation intervention. The results also show that toxigenic, potentially mycoparasitic *Trichoderma atroviride* strains represented the dominant morphotype, as shown in Figure 9D, on the three plates in both extract filter dust and settled dust collected before the ventilation intervention (Table 8). The dominant morphotypes in this dust contained potentially pathogenic *Aspergillus* and toxigenic *Penicillium* species.

In dust collected after the ventilation intervention, the dominant mycobiota (except *Rhizopus* sp.) consisted mainly of nontoxigenic *Penicillium* strains and toxigenic *Aspergillus* species unable to grow at 37 °C (Table 8). One toxigenic *Trichoderma longibrachiatum* strain able to grow at 37 °C, one toxigenic *T. trixiae* and one toxigenic *Penicillium expansum* colony were found on one plate each. Reference samples collected outside of the school contained toxigenic *Curvularia* sp.—like colonies, rendering these outdoor samples the highest number of toxigenic colonies. The hay barn dust did not contain toxigenic species or potentially pathogenic *Aspergillus* strains. The indoor mycobiota differed in species composition from the outdoor one, and changed simultaneously with the ventilation intervention. Interestingly, no mycoparasitic *Trichoderma atroviride* strains were cultivable from the outdoor samples or barn dust. This indicates that this species, isolated from the extract filter and settled dust before the ventilation intervention, may have emanated from an indoor source.

It should be noted that the dust collected from the extract air filter represents the air collected from the studied building part during a several months’ period of time, thus being an exceptionally encompassing sample. However, the filter may have been impacted also by outdoor air if the classroom windows have been kept open, which needs to be considered when assessing the representativeness of the results for indoor air of a given building.

### 3.5. Indoor Air Questionnaire

Indoor air questionnaire results from the studied building section and from the other building sections are presented in Table 9. Occupants answered the questionnaire in the studied building section before the ventilation intervention (May 2016) and after the intervention (January 2017), as well as simultaneously in the other building sections where no ventilation or other changes had taken place. Because of the low amount of data, Fisher’s exact test was used in the statistical analysis of several factors instead of the chi-squared test.

In the studied building section, the known changes in the conditions between the 2016 and 2017 questionnaires were outdoor conditions (seasons) and the ventilation intervention. In other building sections, the only known change was the season. Also, psychosocial factors have an impact on perceptions [25]. In the studied building section, conclusions about the effect of psychosocial factors on IAQ perceptions could not be drawn compared to other building parts.

The first questionnaire (May 2016) was conducted during the spring when pollen may increase the symptoms of allergic diseases and thus effect the perception of indoor climate; however, the air handling unit had effective fine F7 class filters. The second questionnaire (January 2017) was conducted during the winter when the indoor air was very dry because of the low absolute humidity of the outdoor air. Dry air typically increases the prevalence of cough, nose and eye irritation, and skin symptoms [25]. Mechanical ventilation is shown to increase symptom prevalence, related especially to air dryness [2,6,33]. The impact of the season (spring/winter) can be seen from the results of other parts of the school that were not under intervention.

Due to the low number of occupants and the low response rate (58% and 62%) in the studied building section, individual answers were emphasized and significant differences (*p*-value < 0.05) were mostly not found in the statistical analysis (Table 9). The unwanted negative pressure differences, especially during holidays, might have affected indoor conditions and occupants’ perceptions. Between 2016 and 2017, the prevalence of hoarse, dry throat (*p* = 0.118), heavy-feeling head (*p* = 0.183), cough (*p* = 0.321), and headache, as well as difficulties concentrating (*p* = 0.444), increased. Also, the prevalence of varying temperature (*p* = 0.162), draft (*p* = 0.275), and insufficient ventilation (*p* = 0.372) increased.

However, when comparing the changes in the studied building section to changes in the other building sections, a different trend emerged. The perception of low temperature (*p* = 0.345), stuffy air (*p* = 0.085), dry air (*p* = 0.103), and visible dust (*p* = 0.408) increased between 2016 and 2017 in other building sections; however, the increase was not statistically significant. This can be explained by seasonal changes. However, in the studied building section, no worsening in the same factors (*p* = 1.000) was reported.

A decrease in the perceptions of too high temperature (*p* = 0.477) was reported in the studied building section, while in other sections the number of perceptions was already very low in the first place. Perception of unpleasant odor decreased (*p* = 0.638) in the studied section to the same level as it was in the other sections. An increase in the perception of draft (*p* = 0.275, *p* = 0.253) and insufficient ventilation (*p* = 0.342, *p* = 0.352) was reported correspondingly in both sectors.

In the other building sections, tiredness (*p* = 0.077), nose irritation (*p* = 0.085) and dryness of facial skin (*p* = 0.036) increased, while no change in the studied section was seen (*p* = 1.000). The same tendency was seen in the factors which were statistically not significant, i.e., hand skin symptoms (*p* = 0.352) and shortness of breath (*p* = 0.491) increased as well. This is an interesting result considering that, simultaneously, reported asthma decreased (*p* = 0.253) in the other sections but not in the studied section. In addition, the prevalence of hay fever increased (*p* = 0.188) in the studied section, and the perceptions of heavy-feeling head (*p* = 0.183) and cough (*p* = 0.381) also increased more than in the other sections (*p* = 0.706 for both).

It can be concluded that IAQ in the building was perceived as being worse more frequently in 2017. In the studied building section, major improvements in occupants’ perceptions were not reported; however, neither was reported significant worsening in many of the factors that worsened in the other building parts during the follow-up time.

## 4. Conclusions

Ventilation is an essential part of indoor environment quality. As shown in this study, even relatively small changes in air flow rates and pressure differences can have noticeable effects on the measurable and perceivable parameters of indoor air. CO_2_ concentration is often highlighted as an indicator of sufficient ventilation rate, but should never be used alone to determine the state of ventilation in an indoor space. In the studied building, CO_2_ measurements alone would have indicated good IAQ and sufficient ventilation. In order to study IAQ and occupants’ well-being in an indoor space, a holistic ventilation investigation was revealed to be essential.

The aim of 5–7 Pa positive pressure for a period of nine months was only partly reached. In another studied classroom, the pressure difference was mostly close to 0 Pa. The TVOC and PM_2.5_ concentrations decreased significantly after ventilation intervention, yet these were initially very low.

The occupant questionnaire data analysis suffered on the small sample typical for this kind of study and statistically significant improvements were mostly not demonstrated. The results were, however, analyzed to determine possible differences between the studied building section and the other parts of the building, where no actions were taken. In the follow-up questionnaire conducted after five months of positive pressure, the occupants’ perceptions had worsened more moderately, if at all, compared to the occupants’ perceptions in the other building sections, where perceptions had worsened significantly and the only known change in conditions was the season. Seasonal effects on human perceptions were significant due to the cold and dry air of the winter, and also likely due to psychosocial aspects, e.g., darkness during the winter. This effect can be seen in the questionnaire data from the other building sections, but not as clearly in the data from the studied building section. This indicates that positive pressure could be a practical way to decrease perceived IAQ-related problems, but research with a larger occupant sample and studying the same season during the follow-up are needed to confirm this indication.

This study also shows that the change in pressure conditions coincided with a partial but important change in species composition of the indoor microbiome: The potentially pathogenic species of *Aspergillus* able to grow at 37 °C (*Aspergillus* section Nigri and section Flavi strains) were replaced with *Aspergillus* strains unable to grow at 37 °C (*Asp. westerdijkiae* and *Asp. versicolor*-like strains). There are only a few *Aspergillus* species that frequently cause infections in humans: *A. fumigatus* and *A. niger* which are responsible for more than 90% of systemic and lung infections whereas *A. flavus* is the main cause of eye infections [34]. The occurrence of potentially pathogenic isolates of *Paecilomyces* and *Trichoderma* species are of concern. However, these strains seem to be minor representatives of the viable mycobiota.

The occurrence of potentially mycoparasitic *T. atroviride* strains disappeared after the intervention. The small-sized conidia (<4 µm) of *T. atroviride*, *Aspergillu*s, and *Penicillium* species from extract filters collected under negative pressure differences may represent viable fungi emanating from building constructions, as fungal spore penetration in indoor spaces under negative pressure has previously been shown.

The new method used for microbial sampling is proposed for wider testing. In this method, microbes were collected from the extract air filters as representatives of the microbiome within full building and its constructions, which are potentially and occasionally liberated into indoor air due to fluctuations in pressure differences across the building envelope. According to this study, this method could be a relevant way to investigate sources of impurities in buildings with indoor air problems.

This study indicates that by ventilation interventions, conditions in a building with unsolved IAQ problems or awaiting repairs can be controlled without other major temporary actions. Positive pressure could be efficient, especially in a building with microbial damage or other impurities infiltrating from the structures. When utilizing positive pressure, the pressure difference should be high enough to constantly cover all spaces and the variation in ventilation. Potential excess moisture indoors must be low or controlled by ventilation to prevent condensation inside the structures.

## Figures and Tables

**Table 1:** Microbial growth in outer wall structures of Classroom 3 [15].

Room	Place	Date	Fungi (cfu/g)	Bacteria (cfu/g)	Actinobacteria (cfu/g)
Classroom 3	Outer wall, window frame fastening wood	April 2016	170,000 *Aureobasidium* sp., *Mucor* sp., *Penicillium* sp., yeast, sterile	1,400,000	<100 **
Classroom 3	Outer wall, mineral wool in the plinth cut	April 2016	8000 *Exophiala* * sp.	100	29,000 *
Limit value [16]			10,000	100,000	3000

* Moisture damage indicator microbe. ** Below the detection limit 100 cfu/g.

**Table 2:** Measurement methods, devices and their accuracy, measurement place and duration.

Measured Factor	Device	Accuracy	Place	Time
Supply/extract airflow rate	Swemaflow 125D	Air flow: ±3.5%, not better than 0.4 L/s. With backpressure: ±10%, lowest 1 L/s. T ±0.5 °C. Barometer ±3.5 hPa.	Corridor, toilets	Instant
	SWEMA 3000md	±0.3% read value, lowest ±0.3 Pa	Classrooms	60 s average (used to calculate air flow rate)
Pressure difference across the envelope	KIMO CP101, logger Grant 1000	1.5% of reading ±3 Pa	Classrooms 3 and 9	Continuous
Temperature (T)	Rotronic CL11	±0.3 °C	Classrooms 3 and 9	Continuous
Relative humidity (RH)	Rotronic CL11	±3% (10 ... 95%)	Classrooms 3 and 9	Continuous
Carbon dioxide (CO_2_)	Rotronic CL11	±(30 ppm + 5% of reading)	Classrooms 3 and 9	Continuous
T and RH	ThermaData	±0.5 °C (−10 … 85 °C)	Outdoors	Continuous
Formaldehyde	FM-801	±10 ppb at 40, 80, 160 ppb	Classroom 3	Continuous
Carbon monoxide (CO)	TG-501 probe	<4%/year	Classroom 3	Continuous
Particulate matter 2.5 μm (PM_2.5_)	MIE pDR-1500	±5%	Classroom 3	Continuous
Volatile organic compounds (VOC)	Tenax TA, TD-GC-MS	±20% (average)	Classrooms 3 and 9, corridor	40 min
Mycobiota of settled and filter dust			Extract air filter, Classrooms 3 and 9, outdoors	Cultivated for 4 weeks
Perceived indoor air quality	Örebro (MM40)—questionnaire (Finnish Institute of Occupational Health (FIOH))		The occupants of the whole building	2 weeks’ response time

**Table 3:** Supply and extract airflow rates of the studied building section before ventilation balancing, after balancing, and after generating positive pressure.

Air Flow Rates (L/s)	Designed	Measured before Balancing 30 May 2016		Measured after Balancing 22 July 2016		Measured after Overpressure 2 September 2016	
Room	Supply/Extract	Supply	Extract	Supply	Extract	Supply	Extract
1	135/135	146	189	134	135	139	125
2	270/270	313	359	273	273	287	255
3	270/270	273	348	268	265	278	247
4	270/270	301	368	271	271	291	305
5	270/270	283	341	263	264	273	269
6	270/270	259	298	271	271	276	259
7	220/220	141	287	204	202	203	188
8	80/80	126	118	65	62	65	65
9	215/215	215	262	213	216	219	214
10	135/135	133	149	136	134	143	139
11	270/270	289	279	265	264	284	277
12	180/180	175	232	175	175	177	167
Classroom total	2585/2585	2655	3230	2538	2532	2636	2510
Cleaning storage	-/30		13		30		31
Toilet 1	-/30		10		30		32
Toilet 2	-/30		11		30		30
Toilet 3	-/30		13		30		36
Toilet 4	-/30		15		30		34
Toilet 5	-/30		17		30		30
Toilet 6	-/30		17		30		30
Corridor	320/110	330	90	329	114	303	79
TOTAL	2905/2905	2984	3415	2867	2856	2938	2812

**Table 4:** Relative humidity, temperature, and CO_2_ in Classrooms 3 and 9 during school’s occupancy hours, from 8 a.m. to 5 p.m. on Monday–Friday, between 24 August 2016 and 9 May 2017.

	Classroom 3			Classroom 9		
	RH (%)	T (°C)	CO_2_ (ppm)	RH (%)	T (°C)	CO_2_ (ppm)
Average	31	21	518	30	21	520
Min	5	17	391	4	19	382
Max	75	24	972	77	34	1264

**Table 5:** TVOC, VOCs (concentrations over 1 µg/m^3^), formaldehyde, and CO before (a) and after (b) ventilation intervention.

Chemical Compounds of Indoor Air	Classroom 3		Classroom 9		Corridor		Limit Value [29]
(µg/m^3^)	a	b	a	b	a	b	
TVOC	31	19	42	19	34	27	400
Acetic acid		2	1		1	2	50
Acetic acid, butyl ester						2	50
Octamethylcyclotetrasiloxane			4		2	4	50
Decamethylcyclopentasiloxane			4	1	2	3	50
2-ethyl-1-hexanol (2E1H)	2		1		3	3	10
Phenoxyethanol		2				2	50
1-Butanol						1	50
Nonanal	3	1	3	1	3	2	50
Decanal	2	1	2	1	2	2	50
Acetone			1				50
Formaldehyde	*	*	-	-	-	-	50/100
Carbon monoxide (CO)	-	300–1300	-	-	-	-	7000

a: Sample taken on 23 May 2016; b: Sample taken on 2 September 2016. * Below detection limit 10 ppb (~12 µg/m^3^).

**Table 6:** PM_2.5_ concentrations in Classroom 3 before and after the ventilation intervention: Statistical parameters.

PM_2.5_	Before	After
Measurement points	9572	12,569
Mean	1.80	1.27
Standard deviation	1.06	0.97
Min	0.10	0.10
Max	5.90	5.90

**Table 7:** The twelve morphotypes representing the dominant fungal genera and species isolated from the extract filter, settled dust and outdoor air.

	HAMBI Code	Growth at 37 °C	Toxicity		Colony Color	Size of Conidia/Spores	Morphology in Light Microscope
			BMSI	ICP	MEA	(µm)	
*1. Aspergillus* section Flavi	834	+	−	−	green	5–8	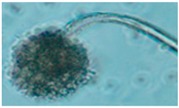
2 strains		+	−	−	green	5–8	
*2. Aspergillus* section Nigri	379	+	−	+	black	3.5–5	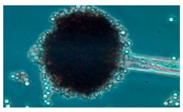
10 strains		+	−	+	black	3.5–5	
*3. Asp. versicolor*	3670	−	−	+	yellow	2–3	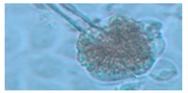
2 strains		−	−	+	yellow	2–3	
*4. Asp. westerdijkiae*	3333	−	+	+	yellow	2.5–3	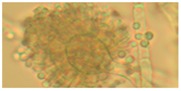
5 strains		−	+	+	yellow	2.5–3	
*5. Curvularia* sp.			+	+	black-grey	15 × 5	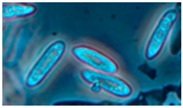
10 strains			+	+	grey	Conidio-phore 15 × 5	
*6. Paecilomyces variotii*	3342	+	+	−	yellow	3–5	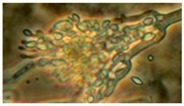
2 strains		+	+	−	yellow	3–5	
*7. Penicillium expansum*	3610	−	+	+	grey	3	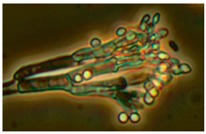
1 strain		−	+	+	grey	3	
*8. Penicillium chrysogenum* *P. commune*	3631	−	−	+	green	3–4	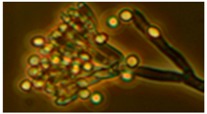
10 strains		−	−	+	green	3–4	
*9. Penicillium* sp.		−	−	−	yellow	3	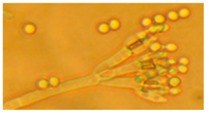
10 strains		−	−	−	yellow	3	
*10. Trichoderma longibrachiatum*	3643	+	+	+	green	1.5 × 3	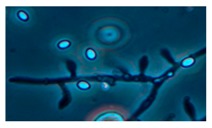
1 strain		+	+	+	green	1.5 × 3	
*11. Trichoderma atroviride* *T. trixiae*	3369	−	+	+	green	3	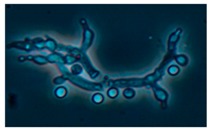
6 strains		−	+	+	green	3	
*12. Rizopus* sp.		−	−	−	black	Spores 7–10	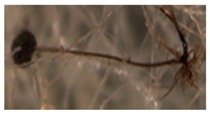
10 strains		−	−	−	black	Spores 7–10	

**Table 8:** Cultivable mycobiota in indoor dust from the extract air filter and from classroom surfaces above the floor level. The dust was sampled before and after the ventilation intervention. Names in brackets indicate the presence of a single colony on a single plate.

	Microbial Genera by Sampling Month		Toxic Colonies (%)	Number of Plates Containing Colony Morphotype/All Plates
School samples	Settled dust	Extract air filter	Settled dust + Extract air filter	
Before ventilation intervention	23 May 2016	28 August 2016	80	
	*Rhizopus* sp.	*Rhizopus* sp.		3/10
	*T. atroviride* *	*T. atroviride* *		3/10
		*Penicillium* *chrysogenum* * P.commune *		7/10
		*Aspergillus niger* *^,1^		7/10
		*Aspergillus flavus* ^1^		2/10
		(*Paecilomyces* sp.) *^,1^		1/10
	*Asp. Versicolor* *			2/10
After ventilation intervention	2 September 2016	6 February 2017	40	
	*Rhizopus* sp.	*Rhizopus* sp.		6/12
	*Penicillium* sp.	*Penicillium* sp.		10/12
		*Asp. Westerdijkiae* *		3/12
		(*Penicillium expansum*) *		1/12
	(T. trixiae *)			1/12
		(*T. longibrachiatum*) *^,1^		1/12
Reference samples (outdoors)	Fall out plate	Settled dust		
Sampling date	28 August 2016	28 August 2016		
School yard	*Curvularia* sp.*	*Curvularia* sp.*	90	4/6
Barn dust	*Penicillium* sp.	*Penicillium* sp.	<10	6/6

^1^ Potential pathogens able to grow at 37 °C. * Toxigenic when screened with boar sperm inhibition test and/or cytotoxicity against porcine kidney cells.

**Table 9:** Indoor climate questionnaire (Finnish Institute of Occupational Health^©^ 2006–2008, version 2.0) results from spring 2016 and winter 2017. Statistically significant changes at a 10% confidence interval (*p* < 0.1) are bolded. *p*-values marked with * were determined by the Fisher’s exact test (SPSS). Comparison values are based on the analysis of comprehensive questionnaire data collected by FIOH.

	Comparison Values	Studied Part		*p*-Value	Other Parts		*p*-Value
	[30,31,32]	2016	2017		2016	2017	
**Background information**							
Number of answers		10	8		28	29	
Answer (%)	71	58	62		65	72	
Females (%)	21	60	75		75	86	
Daily smokers (%)		10	0		0	0	
Average age (years)		46	45		44	46	
Average employment in this work place (years)		8	6		10	10	
**Work environment (%)**							
Draught	22	10	38	0.275 *	7	21	0.253 *
Room temperature too high	17	20	0	0.477 *	7	7	1.000 *
Varying temperature	16	20	57	0.162 *	11	22	0.295 *
Room temperature too low	13	30	25	1.000 *	15	25	0.345
Stuffy air	34	30	38	1.000 *	46	69	0.085
Dry air	35	40	50	1.000 *	22	43	0.103
Insufficient ventilation	32	30	63	0.342 *	43	55	0.352
Smell of mold	9	20	25	1.000 *	14	21	0.730 *
Unpleasant odor	17	40	25	0.638 *	26	28	0.889
Environmental tobacco smoke	4	0	0	-	0	0	-
Noise	17	20	25	1.000 *	39	45	0.672
Dim light or reflections	14	10	0	1.000 *	7	10	1.000 *
Dust or dirt	25	40	38	1.000 *	32	43	0.408
**Work regarded as interesting and stimulating (%)**							
Often	75	100	100		89	86	
Sometimes	20	0	0		11	14	
Seldom or never	4	0	0	-	0	0	1.000 *
**Too much work to do (%)**							
Often	20	10	0		14	21	
Sometimes	59	60	75		61	72	
Seldom or never	21	30	25	1.000 *	25	7	0.185 *
**Opportunity to influence work conditions (%)**							
Often	35	60	50		46	38	
Sometimes	44	30	50		46	55	
Seldom or never	21	10	0	0.798 *	7	7	0.839 *
**Fellow workers help with problems in the work (%)**							
Often	72	80	100		82	83	
Sometimes	22	20	0		14	17	
Seldom or never	6	0	0	0.477 *	4	0	1.000 *
**Allergic diseases (%)**							
Asthma	8	10	13	1.000 *	18	7	0.253 *
Hay fever	38	40	75	0.188 *	46	45	0.903
Atopic eczema	28	40	38	1.000 *	14	17	1.000 *
**Stress (%)**							
Quite or very much	10	20	0		18	28	
Some	28	20	63		29	41	
No/only little	63	60	38	0.214 *	54	31	0.226
**Symptoms (%)**							
Fatigue	16	10	13	1.000 *	14	34	0.077
Heavy-feeling head	9	0	25	0.183 *	11	17	0.706 *
Headache	7	0	13	0.444 *	11	18	0.705 *
Concentrating difficulties	3	0	13	0.444 *	4	14	0.352 *
Eye irritation	17	20	13	1.000 *	37	41	0.740 *
Irritated, stuffy or running nose	20	20	25	1.000 *	39	62	0.085
Hoarse/dry throat	14	10	50	0.118 *	36	45	0.483
Cough	5	20	50	0.321 *	11	17	0.706
Cough disturbing sleep	1	10	13	1.000 *	0	0	-
Dry of flushed facial skin	11	10	13	1.000 *	7	29	0.036
Hands: dry, itching, red skin	15	10	0	1.000 *	4	14	0.352 *
Shortness of breath	3	10	13	1.000 *	0	7	0.491 *
Wheezing	1	10	13	1.000 *	0	0	-
Fever or chills	2	0	0	-	4	7	1.000 *
Joint pain	3	10	0	1.000 *	4	0	0.491 *
Muscular pain	4	0	0	-	0	0	-
Other		20	25	1.000 *	4	3	1.000 *

## Abbreviations

The following abbreviations are used in this manuscript:

IAQ	Indoor air quality
TVOC	Total volatile organic compounds
PM_2.5_	Fine particulate matter, particle size 2.5 μm
RH	Relative humidity
VOC	Volatile organic compounds
T	Temperature
CO_2_	Carbon dioxide
CO	Carbon monoxide
FIOH	Finnish Institute of Occupational Health
MEA	Malt extract agar
2E1H	2-ethyl-1-hexanol
